# Evolutionary Conservation and Divergence of Genes Encoding 3-Hydroxy-3-methylglutaryl Coenzyme A Synthase in the Allotetraploid Cotton Species *Gossypium hirsutum*

**DOI:** 10.3390/cells8050412

**Published:** 2019-05-03

**Authors:** Wei Liu, Zhiqiang Zhang, Wei Zhu, Zhongying Ren, Lin Jia, Wei Li, Zongbin Ma

**Affiliations:** 1 Collaborative Innovation Center of Henan Grain Crops/Agronomy College, Henan Agricultural University, Zhengzhou 450002, China; liuwei0205@henau.edu.cn (W.L.); 13598838901@163.com (Z.Z.); zhuwei_2006z@126.com (W.Z.); cathylin2012@163.com (L.J.); 2 State Key Laboratory of Cotton Biology/Institute of Cotton Research, Chinese Academy of Agricultural Sciences, Anyang 455000, China; renzhongying@caas.cn

**Keywords:** *Gossypium*, polyploidization, HMGS, duplicated genes, expression divergence, evolution

## Abstract

Polyploidization is important for the speciation and subsequent evolution of many plant species. Analyses of the duplicated genes produced via polyploidization events may clarify the origin and evolution of gene families. During terpene biosynthesis, 3-hydroxy-3-methylglutaryl coenzyme A synthase (HMGS) functions as a key enzyme in the mevalonate pathway. In this study, we first identified a total of 53 *HMGS* genes in 23 land plant species, while no *HMGS* genes were detected in three green algae species. The phylogenetic analysis suggested that plant *HMGS* genes may have originated from a common ancestral gene before clustering in different branches during the divergence of plant lineages. Then, we detected six *HMGS* genes in the allotetraploid cotton species (*Gossypium hirsutum*), which was twice that of the two diploid cotton species (*Gossypium raimondii* and *Gossypium arboreum*). The comparison of gene structures and phylogenetic analysis of *HMGS* genes revealed conserved evolution during polyploidization in *Gossypium*. Moreover, the expression patterns indicated that six *GhHMGS* genes were expressed in all tested tissues, with most genes considerably expressed in the roots, and they were responsive to various phytohormone treatments and abiotic stresses. The sequence and expression divergence of duplicated genes in *G. hirsutum* implied the sub-functionalization of *GhHMGS1A* and *GhHMGS1D* as well as *GhHMGS3A* and *GhHMGS3D*, whereas it implied the pseudogenization of *GhHMGS2A* and *GhHMGS2D*. Collectively, our study unraveled the evolutionary history of *HMGS* genes in green plants and from diploid to allotetraploid in cotton and illustrated the different evolutionary fates of duplicated *HMGS* genes resulting from polyploidization.

## 1. Introduction

Polyploidization (or whole-genome duplication, WGD) is a significant speciation mechanism and a major driving force in plant evolution [1]. The frequency of polyploidization is high in plants. The highest frequency of polyploidization is exhibited in pteridophytes [2,3], and it is estimated that more than 70% of angiosperms have undergone at least one polyploidization event during their evolutionary history [4]. Whole-genome sequencing analyses have revealed that even plants with relatively small genomes, such as *Arabidopsis thaliana*, have experienced polyploidization events during evolution [5]. The most obvious consequence of polyploidization is the duplicated genes in the genome [6]. Gene duplication is considered to play an important role in acquiring new genes and providing raw materials for the evolution of genetic diversity [7]. Many new gene functions have evolved through gene duplication, which has significantly contributed to the expansion of gene families and the evolution of developmental programs in various organisms [8,9]. Therefore, studying the subsequent fate of the duplicated genes may clarify the evolution of polyploidy. After gene duplication, one copy retains its original function, while the genetic selection of the other copy is relaxed, allowing divergence between duplicated genes [10]. There are theoretically three evolutionary outcomes of duplicated genes: neo-functionalization, sub-functionalization, and pseudogenization [11].

Cotton is a source of renewable textile fiber, making it an economically valuable crop [12]. The genus *Gossypium* includes five tetraploid (2n = 4x = 52) and more than 45 diploid (2n = 2x = 26) species [13]. All of the diploid species have diverged from a common eudicot progenitor approximately 5–10 million years ago [14]. Approximately 1–2 million years ago, an interspecific hybridization occurred between the diploid cotton species resembling *Gossypium arboreum* (AA) and *Gossypium raimondii* (DD), which produced the allotetraploid cotton species, including *Gossypium hirsutum* (A_t_A_t_D_t_D_t_; where ‘t’ stands for tetraploid) [15]. The complete genome sequences of *G. hirsutum* acc. TM-1 [16,17,18] and two diploid species, *G. raimondii* [19,20] and *G. arboreum* [21,22], have provided raw information for evolutionary and functional genomics studies in cotton. *Gossypium* species have developed into an ideal plant for studying genome evolution and plant polyploidization [14,23].

Terpenes are the largest group of natural compounds that are widespread in nature, encompassing at least 50,000 known products in extant organisms [24]. Plant terpenes are functionally diverse, serving as photosynthetic pigments, hormones, electron carriers, major structural membrane components, and phytoalexins [25,26]. Isopentenyl diphosphate (IPP) and its isomer dimethylallyl diphosphate (DMAPP) are the common precursors required for terpene biosynthesis, and are mainly synthesized by two independent pathways in plants, namely, the cytosolic mevalonate (MVA) pathway and the plastidial 2-*C*-methyl-D-erythritol 4-phosphate (MEP) pathway [27,28]. As the second enzyme in the MVA pathway, 3-hydroxy-3-methylglutaryl coenzyme A synthase (HMGS; EC 2.3.3.10) catalyzes the conversion of acetoacetyl-CoA to 3-hydroxy-3-methylglutaryl-CoA (HMG-CoA) [29]. The HMG-CoA is reduced to mevalonate by HMG-CoA reductase (HMGR; EC 1.1.1.34), after which the mevalonate is converted to IPP and DMAPP [30]. Originally, HMGR was considered to be a rate-limiting enzyme within the MVA pathway, and studies have focused on this enzyme [31]. However, there is accumulating evidence suggesting that HMGS also has important regulatory functions [32]. To date, *HMGS* genes have been isolated and cloned from several plants, including *A. thaliana* [33], *Brassica juncea* [34], *Ginkgo biloba* [35], and *Zea mays* [36]. Studies have revealed that *HMGS* genes are important for plants’ resistance to biotic and abiotic stresses, and are also related to the synthesis of plant terpenes. In *G. biloba*, the expression of *GbHMGS1* is reportedly induced by abiotic stresses (ultraviolet B and cold) and hormone treatments (salicylic acid, methyl jasmonate, and ethephon) [35]. A previous study confirmed that the overexpression of *GlHMGS* in *Ganoderma lucidum* (Ling-zhi) resulted in ganoderic acid contents that were approximately 15.1–24.2% higher than the control group [37].

In this study, we performed a genome-wide identification and evolutionary analysis of the *HMGS* genes in various green plants. Our analyses focused on the identification, evolutionary relationships, exon–intron structures, and the chromosomal localization of cotton *HMGS* genes, as well as the expression patterns of duplicated genes in diverse tissues and in response to various stresses in the allotetraploid cotton species. Our results broaden the understanding of the biological function and evolution of cotton *HMGS* genes.

## 2. Materials and Methods

### 2.1. Identification of HMGS Proteins in *Gossypium* and Other Species

The genome data of *G. raimondii* [19], *G. arboreum* [22], and *G. hirsutum* [18] were downloaded from the CottonGen database (https://www.cottongen.org/). The genome data sources of other species analyzed in this study are listed in Table S1. Then, the local blast database was established for the nucleic acid and protein sequences based on these genome data, respectively. The *HMGS* genes were identified using BlastP and tBlastN programs, with *Arabidopsis* HMGS protein sequence [38] retrieved from TAIR10 (https://www.arabidopsis.org/) as the query. Finally, to verify the results, the Pfam [39] and the InterPro [40] databases were used to confirm each candidate *HMGS* gene.

### 2.2. Phylogenetic Analysis, Gene Structure and Chromosomal Mapping

Multiple sequence alignments of HMGS proteins were carried out using the ClustalX software (Version 2.1, Conway Institute UCD, Dublin, Ireland) with default parameters [41]. Full-length plant HMGS proteins were used to construct phylogenetic trees with the maximum likelihood (ML) method of the Jones–Taylor–Thornton (JTT) model in the PhyML software (Version 3.0, University of Montpellier, Montpelier, France) [42] as well as the Neighbor-Joining (NJ) method of the MEGA software (Version 5.2, Biodesign Institute, Tempe, AZ, USA) [43], and the statistical reliability was assessed by a bootstrap test with 1000 replicates. In addition, the NJ method in the MEGA software was also used to construct a phylogenetic tree for *Gossypium* HMGS proteins. The trees were visualized with the FigTree software (Version 1.4.3, University of Edinburgh, Edinburgh, UK).

The exon–intron structures of *Gossypium HMGS* genes were analyzed by comparing the genomic sequences and their corresponding coding sequences using the online Gene Structure Display Server (GSDS) program (http://gsds.cbi.pku.edu.cn/) [44]. Mapping of *HMGS* genes was performed using the MapInspect software (Ralph van Berloo, Wageningen, Netherlands) [45] according to their starting positions on chromosomes searched in the cotton genome database.

### 2.3. Plant Materials and Treatments

*Gossypium hirsutum* acc. TM-1 was used for gene expression analyses involving various tissues. The roots, stems, cotyledons, and leaves were collected from 2-week-old seedlings grown in a greenhouse. Petals were collected from plants grown in an experimental field on the day of flowering, and ovules were collected at 0, 10, 20, 30, and 40 days post anthesis (DPA). All tissues were immediately flash frozen in liquid nitrogen and stored at −80 °C prior to being analyzed.

*Gossypium hirsutum* acc. TM-1 was used for analyzing gene expression levels in response to various phytohormone treatments and abiotic stresses. Cotton seeds were sown in sand and incubated for about 12 days at 28 °C with a 16 h light/8 h dark photoperiod. The seedlings were then transferred to a liquid culture medium, and seedlings with the third true leaf appeared were used for the following treatments. For phytohormone treatments, seedlings were irrigated with 100 μM gibberellin (GA), auxin (IAA), salicylic acid (SA), or methyl jasmonate (MeJA), after which the roots were harvested at 0, 0.5, 1, 3, and 5 h. For the salt and drought treatments, seedlings were irrigated with 150 mM NaCl and 20% polyethylene glycol (PEG) 6000, respectively. For the heat and cold stresses, seedlings were incubated at 38 °C or 4 °C. The leaves were sampled at 0, 1, 3, 6, and 12 h. All collected samples were stored at −80 °C after quick-freezing in liquid nitrogen.

### 2.4. RNA Isolation, cDNA Synthesis, and Primer Design

Total RNA was extracted from cotton samples with the RNA Extraction Kit (TIANGEN, Beijing, China) according to the manufacturer’s protocol. A NanoDrop2000 microvolume spectrophotometer (Thermo Fisher Scientific, Wilmington, DE, USA) was used to measure the RNA concentration, while the integrity of RNA was assessed by 1.5% agarose gel electrophoresis. First-strand cDNA was synthesized from 1 μg total RNA with the PrimeScript™ 1st Strand cDNA Synthesis Kit (TaKaRa, Dalian, China). Gene-specific primers were designed based on the coding sequences using the Oligo software (Version 7.0, Molecular Biology Insights, Cascade, CO, USA) [46], and then synthesized by the Suzhou GENEWIZ company (Tables S2 and S3). 

### 2.5. Reverse Transcription PCR (RT-PCR) and Quantitative Real-Time RT-PCR (qRT-PCR)

The RT-PCR reactions were completed with Tks Gflex™ DNA Polymerase (TaKaRa) and the following program: 94 °C for 1 min; 35 cycles of 98 °C for 10 s, 60 °C for 15 s, and 68 °C for 1 min. The amplified fragments were purified with the MiniBEST Agarose Gel DNA Extraction Kit (TaKaRa), cloned into the pMD18-T cloning vector (TaKaRa), and inserted into *Escherichia coli* DH5α cells. At least eight clones per gene were randomly selected and sequenced.

The qRT-PCR reactions were performed on the LightCycler480 system (Roche, Basel, Switzerland) using SYBR^®^ Premix Ex Taq™ (TaKaRa). The cotton *UBQ7* gene was used as an internal control. The amplification program was as follows: 95 °C for 5 min; 40 cycles at 95 °C for 10 s, 60 °C for 10 s, and 72 °C for 10 s; for the melting curve stage, the default settings were chosen. Because the *HMGS* genes showed high sequence similarity, the specificity of all qRT-PCR primer pairs was verified by RT-PCR to distinguish individual genes. Eight clones per amplified product of each primer pair were randomly selected for sequencing to confirm primer specificity. The relative expression levels of *HMGS* genes were calculated according to the comparative cycle threshold (Ct) method [47]. The results were performed using the OriginPro software (Version 8.0, OriginLab Corporation, Northampton, MA, USA) [48].

## 3. Results

### 3.1. Identification and Phylogenetic Analysis of HMGS Genes in Green Plants

**Figure 1:**
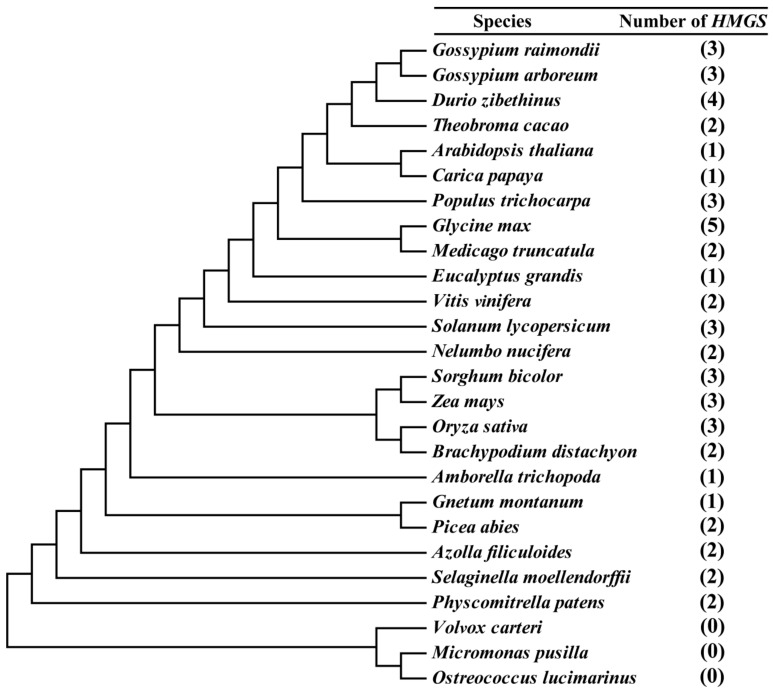
Inferred phylogenetic relationships among 26 species. The number of *HMGS* genes detected in each genome is indicated on the right.

To identify the *HMGS* genes in green plants, we searched for *HMGS* genes in *G. raimondii* [19], *G. arboreum* [22], and 24 other species with published genome-wide data. These 24 additional species represented eight plant evolutionary lineages. Specifically, the analyzed species included green algae (*Ostreococcus lucimarinus*, *Micromonas pusilla*, and *Volvox carteri*), a bryophyte (*Physcomitrella patens*), a lycophyte (*Selaginella moellendorffii*), a pteridophyte (*Azolla filiculoides*), gymnosperms (*Gnetum montanum* and *Picea abies*), a basal angiosperm (*Amborella trichopoda*), monocots (*Brachypodium distachyon*, *Oryza sativa*, *Z. mays*, and *Sorghum bicolor*), a basal eudicot (*Nelumbo nucifera*), and core eudicots (*Solanum lycopersicum*, *Vitis vinifera*, *Eucalyptus grandis*, *Medicago truncatula*, *Glycine max*, *Populus trichocarpa*, *Carica papaya*, *A. thaliana*, *Theobroma cacao*, and *Durio zibethinus*). Candidate HMGS proteins identified from these genomes using the local blast program were submitted to the Pfam protein families database and analyzed for the presence of conserved HMG_CoA_synt_C (PF08540) and HMG_CoA_synt_N (PF01154) domains. Then, the putative HMGS proteins were compared with the sequences in the InterPro database, which revealed the proteins belonged to the HMGS family (IPR010122). The *HMGS* genes identified in these species were named with a species-specific letter as a prefix and a numerical suffix, which was based on the chromosomal position of the gene (Table S4). The evolutionary relationships among the green plants and the number of corresponding *HMGS* genes were determined (Figure 1). The *HMGS* gene family evolutionarily first appeared in the bryophyte *P. patens*, implying these genes arose as plants transitioned from water to land. Additionally, the size of the *HMGS* gene family varied from one to five copies. Five species (*G. montanum*, *A. trichopoda*, *E. grandis*, *C. papaya*, and *A. thaliana*) carried a single copy, *G. max* contained five copies, *D. zibethinus* contained four copies, and most of the other species had two or three copies.

**Figure 2:**
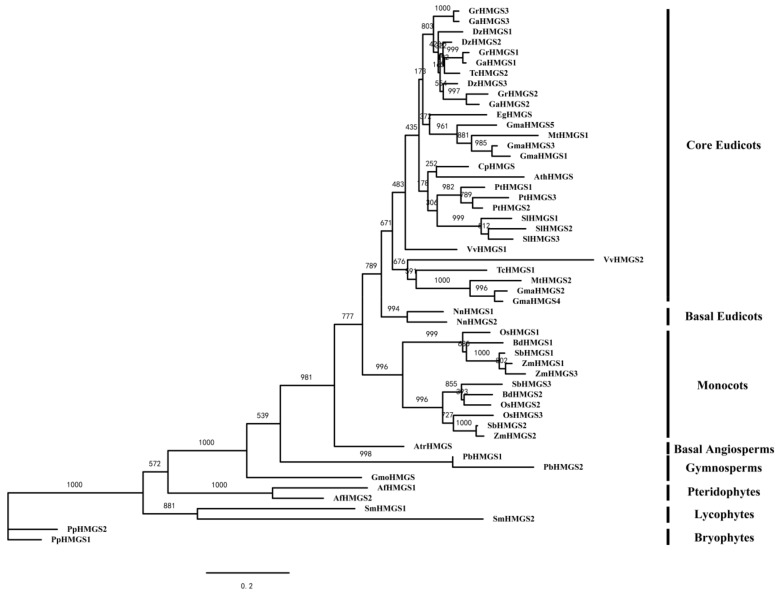
Phylogenetic tree based on HMGS proteins from *G. raimondii*, *G. arboreum*, and 24 other species. The phylogenetic tree was constructed with the ML method of the JTT model in the PhyML software. The bootstrap analysis was conducted with 1000 replicates.

To further investigate the evolutionary relationships among HMGS proteins in green plants, a ML phylogenetic tree was constructed with the PhyML software, and a NJ phylogenetic tree was built with MEGA software based on full-length HMGS protein sequences (Figure 2 and Figure S1). The two phylogenetic trees showed similar topologies with only minor modifications. The *HMGS* genes of flowering plants were divided into two large subclades (i.e., monocots and eudicots). The *HMGS* genes derived from monocots and eudicots did not cluster together in the phylogenetic trees, suggesting that the plant *HMGS* genes may have originated from the same ancestral gene and subsequently differentiated as the plant lineages diverged. There were several unique evolutionary branches in the phylogenetic trees, such as *S. lycopersicum*, *P. trichocarpa*, and *G. max*, indicating that after these species formed, the *HMGS* gene families may have undergone species-specific expansions, which increased the number of *HMGS* genes in their genomes.

### 3.2. Phylogenetic Analysis of Gossypium HMGS Genes

**Figure 3:**
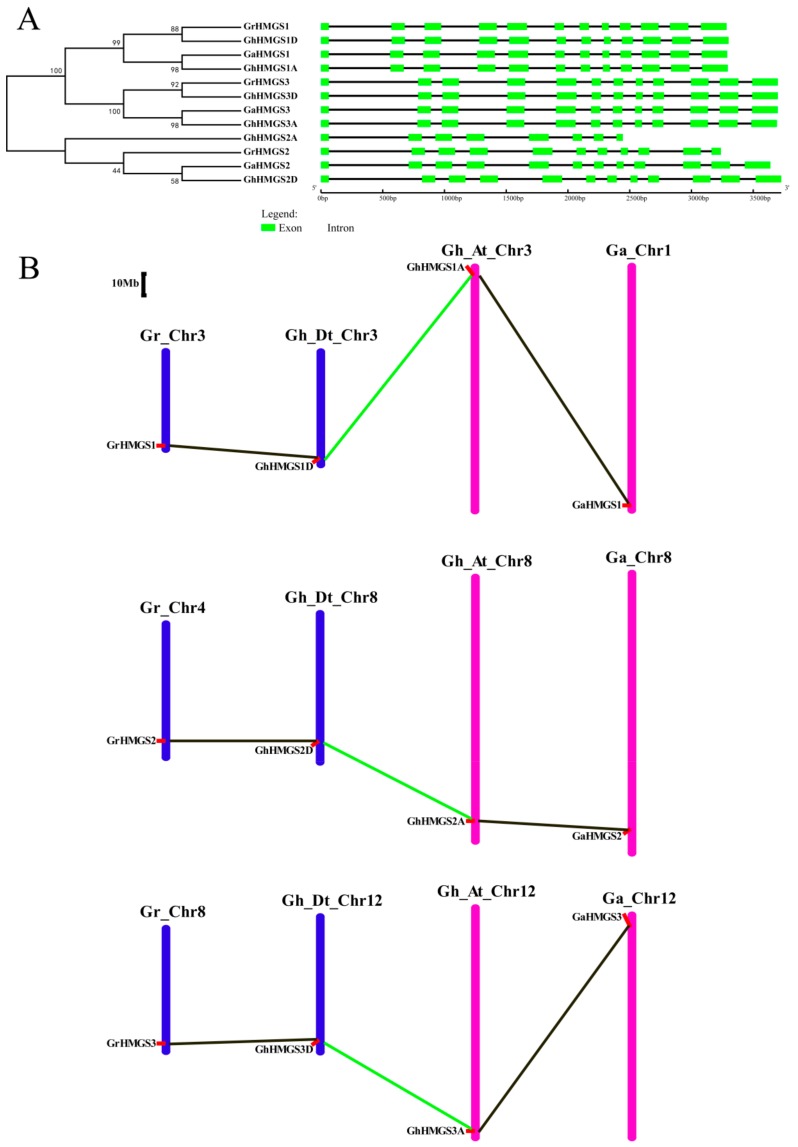
Phylogenetic relationships, gene structures, and chromosomal distributions of *Gossypium HMGS* genes. (**A**) Phylogenetic relationships and gene structures of *HMGS* genes from *G. raimondii*, *G. arboreum*, and *G. hirsutum*. Exons and introns are represented by green boxes and black lines, respectively. (**B**) Chromosomal distributions of *HMGS* genes from *G. raimondii*, *G. arboreum*, and *G. hirsutum*. Chromosome numbers are displayed at the top of each bar. The chromosomes of *G. raimondii* and the *G. hirsutum* D-subgenome are indicated in blue, while the chromosomes of *G. arboreum* and the *G. hirsutum* A-subgenome are indicated in magenta. The putative orthologous *HMGS* genes between the two diploid species and the allotetraploid species are connected by black lines, and the putative paralogous *HMGS* genes between the D-subgenome and A-subgenome of *G. hirsutum* are connected by green lines.

We identified *HMGS* genes in the allotetraploid cotton species, *G. hirsutum*. All of the putative coding sequences of *Gossypium HMGS* genes were re-predicted with FGENESH [49] and then verified by gene cloning and sequencing. Consequently, three, three, and six *HMGS* genes were identified in *G. raimondii*, *G. arboreum*, and *G. hirsutum*, respectively. The *G. hirsutum HMGS* genes were named according to their homologous relationships with *G. raimondii* and *G. arboreum* genes, and the suffixes D and A were added to represent the subgenome (Table S5). Particularly, gene sequencing results and sequence alignments indicated that *GrHMGS2* from *G. raimondii* and *GhHMGS2A* from *G. hirsutum* may be pseudogenes that do not encode functional proteins because of a frameshift mutation caused by a base insertion (Figure S2). Additional sequence alignments showed that all the *Gossypium* HMGS proteins contained a conserved motif “NxD/NE/VEGI/VDx(2)NACF/YxG”, which is important for HMGS function. Furthermore, they all had five conserved active sites (amino acids Glu83, Cys117, Ser248, Gly325, and Ser359), except *GhHMGS2A* (Figure S3). To gain further insights into the evolution of gene structures, the exon–intron structure of the *Gossypium HMGS* genes was analyzed (Figure 3A). Except for the two pseudogenes, all of the *HMGS* genes shared the same exon–intron organization with 12 exons and 11 introns and had almost the same exon lengths. The *GrHMGS2* and *GhHMGS2A* structures (i.e., 11 and 8 exons, respectively) differed because of the presence of a premature termination codon. Chromosomal localizations showed that three *GrHMGS* genes, *GrHMGS1*, *GrHMGS2*, and *GrHMGS3*, were present on chromosomes 3, 4, and 8, respectively. Three *GaHMGS* genes, *GaHMGS1*, *GaHMGS2*, and *GaHMGS3*, were located on chromosomes 1, 8, and 12, respectively. In *G. hirsutum*, *GhHMGS1D*, *GhHMGS2D*, and *GhHMGS3D* were separately localized to chromosomes 3, 8, and 12 of the D-subgenome. Meanwhile, in the A-subgenome, *GhHMGS1A*, *GhHMGS2A*, and *GhHMGS3A* were detected on chromosomes 3, 8, and 12, respectively. The *HMGS* genes in the two diploid species and in the two subgenomes of the allotetraploid species had a corresponding homologous relationship. Additionally, there were three pairs of duplicated genes resulting from polyploidization in the allotetraploid species (Figure 3B).

### 3.3. Expression Profiles of HMGS Genes in Various G. hirsutum Tissues

**Figure 4:**
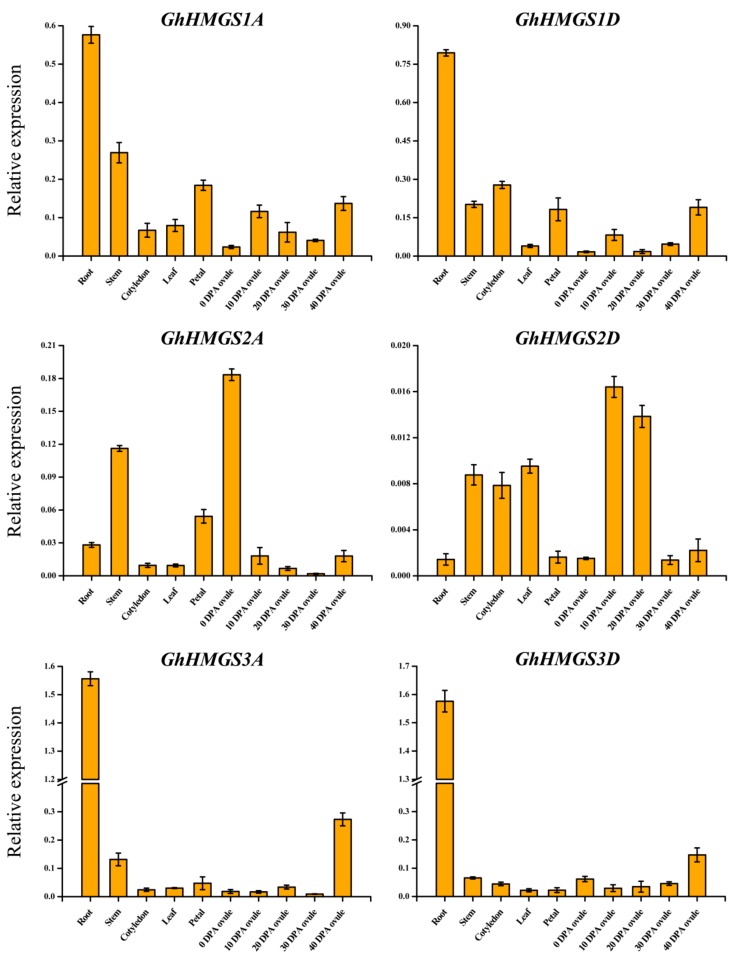
Expression analysis of *G. hirsutum HMGS* genes in various tissues. The expression patterns of *GhHMGS* genes were detected in the roots, stems, cotyledons, leaves, petals, and ovules (0, 10, 20, 30, and 40 DPA) and calculated with the cotton *UBQ7* gene as an internal control. Error bars represent the standard deviations estimated from three independent replicates.

To explore the possible functions of *HMGS* genes, we analyzed the expression profiles of *HMGS* genes in various tissues of the cultivated allotetraploid cotton species *G. hirsutum* acc. TM-1, including the roots, stems, cotyledons, leaves, petals, and developmental ovules (0, 10, 20, 30, and 40 DPA). The *HMGS* genes were expressed in all examined tissues and exhibited tissue-specific expression patterns (Figure 4). Specifically, *GhHMGS1A* and *GhHMGS1D* were most highly expressed in the roots. Additionally, *GhHMGS1A* was moderately expressed in the stems, petals, and ovules at 10 and 40 DPA. In contrast, *GhHMGS1D* was moderately expressed in the stems, cotyledons, petals, and ovules at 40 DPA, but was only slightly expressed in the ovules at 0 and 20 DPA. Considerably different expression patterns were observed between *GhHMGS2A* (a pseudogene) and its paralog in the D-subgenome, *GhHMGS2D*. Although the *GhHMGS2A* and the *GhHMGS2D* expression levels were generally low in the examined tissues, *GhHMGS2A* expression was relatively high in the ovules at 0 DPA and stems, whereas *GhHMGS2D* expression was relatively high in the ovules at 10 and 20 DPA. Meanwhile, the *GhHMGS3A* and *GhHMGS3D* expression patterns were similar. Both genes were highly expressed in the roots and ovules at 40 DPA. Furthermore, *GhHMGS3A* was also relatively highly expressed in the stems, petals, and ovules at 20 DPA, and *GhHMGS3D* was also significantly expressed in the stems, cotyledons, and ovules at 0 and 30 DPA.

### 3.4. Expression Analysis of HMGS Genes in Response to Phytohormone Treatments

**Figure 5:**
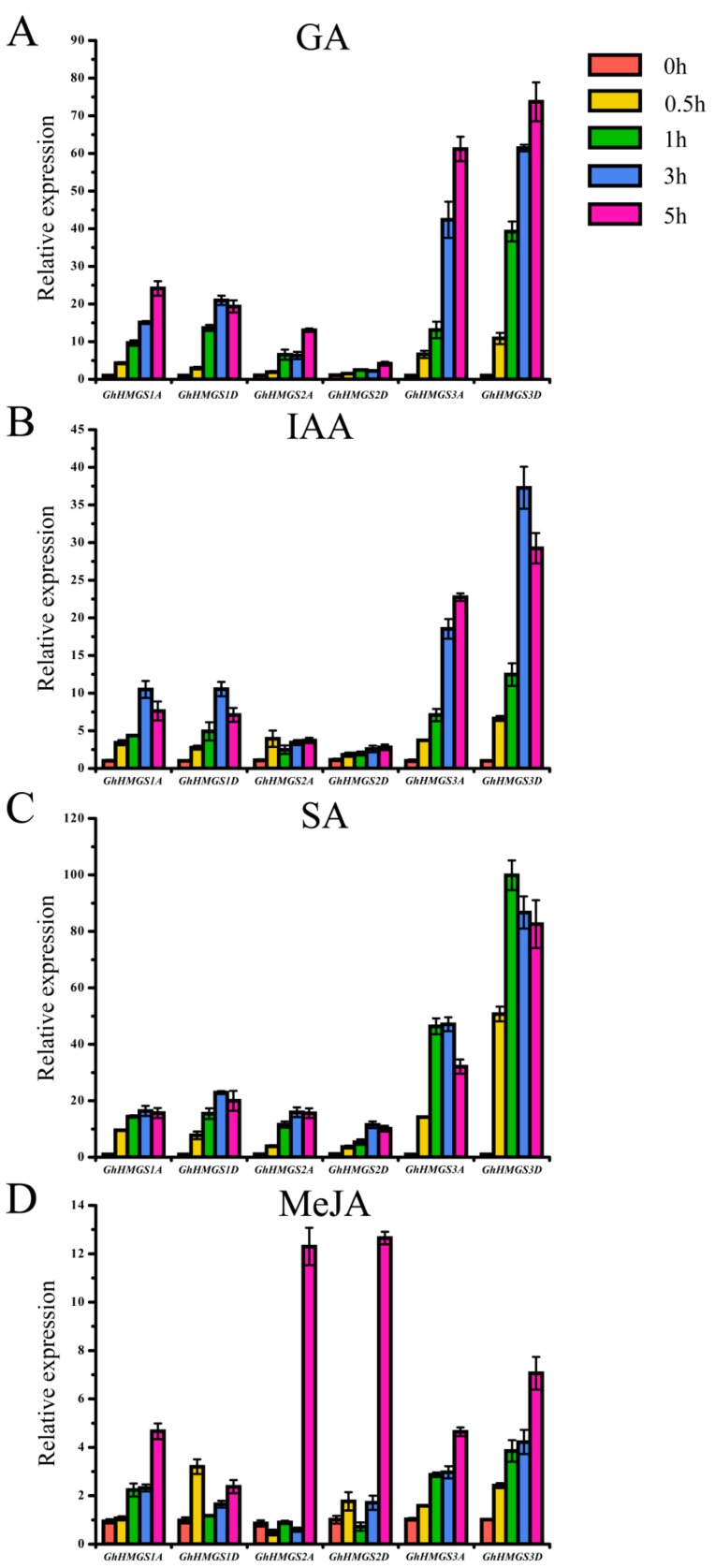
Expression analysis of *G. hirsutum HMGS* genes under phytohormone treatments. (**A**) GA; (**B**) IAA; (**C**) SA; (**D**) MeJA. The relative expression levels of *GhHMGS* genes under different phytohormone treatments were calculated with the cotton *UBQ7* gene as an internal control. Error bars represent the standard deviations estimated from three independent replicates.

Previous studies have concluded that the expression of *HMGS* genes could be up-regulated by methyl jasmonate (MeJA) and salicylic acid (SA) in *Chamaemelum nobile* [50] and by abscisic acid (ABA) in *G. lucidum* (Ling-zhi) [37]. To analyze *GhHMGS* expression levels in response to various phytohormones, we determined the *GhHMGS* expression profiles under gibberellin (GA), auxin (IAA), SA, and MeJA treatments (Figure 5). Both *GhHMGS1A* and *GhHMGS1D* were strongly induced by GA, IAA, and SA treatments, but were up-regulated by MeJA to varying degrees. The expression level of *GhHMGS1A* increased over time following the MeJA treatment, but *GhHMGS1D* expression peaked at 0.5 h after the MeJA treatment and then decreased to the control level, followed by another increase. *GhHMGS2D* was induced by all phytohormone treatments to varying extents. For example, *GhHMGS2D* was strongly induced by SA and was only slightly up-regulated under the GA and IAA treatment. In response to MeJA, the transcript level of *GhHMGS2D* underwent relatively minimal changes during the initial time points but was significantly increased at 5 h. The *GhHMGS2D* paralog in the A-subgenome, *GhHMGS2A*, which is a pseudogene with a premature stop codon in its coding sequence, produced an expression pattern that was similar to that of *GhHMGS2D* in response to GA, IAA, and SA. However, under the MeJA treatment, *GhHMGS2A* expression level was slightly down-regulated over the first 3 h before significantly increasing at 5 h. *GhHMGS3A* and *GhHMGS3D* were strongly induced by all four phytohormone treatments. Additionally, their expression profiles were very similar, although *GhHMGS3A* expression was up-regulated after the IAA treatment, whereas *GhHMGS3D* expression peaked at 3 h and then decreased slightly.

### 3.5. Expression Analysis of HMGS Genes in Response to Abiotic Stresses

**Figure 6:**
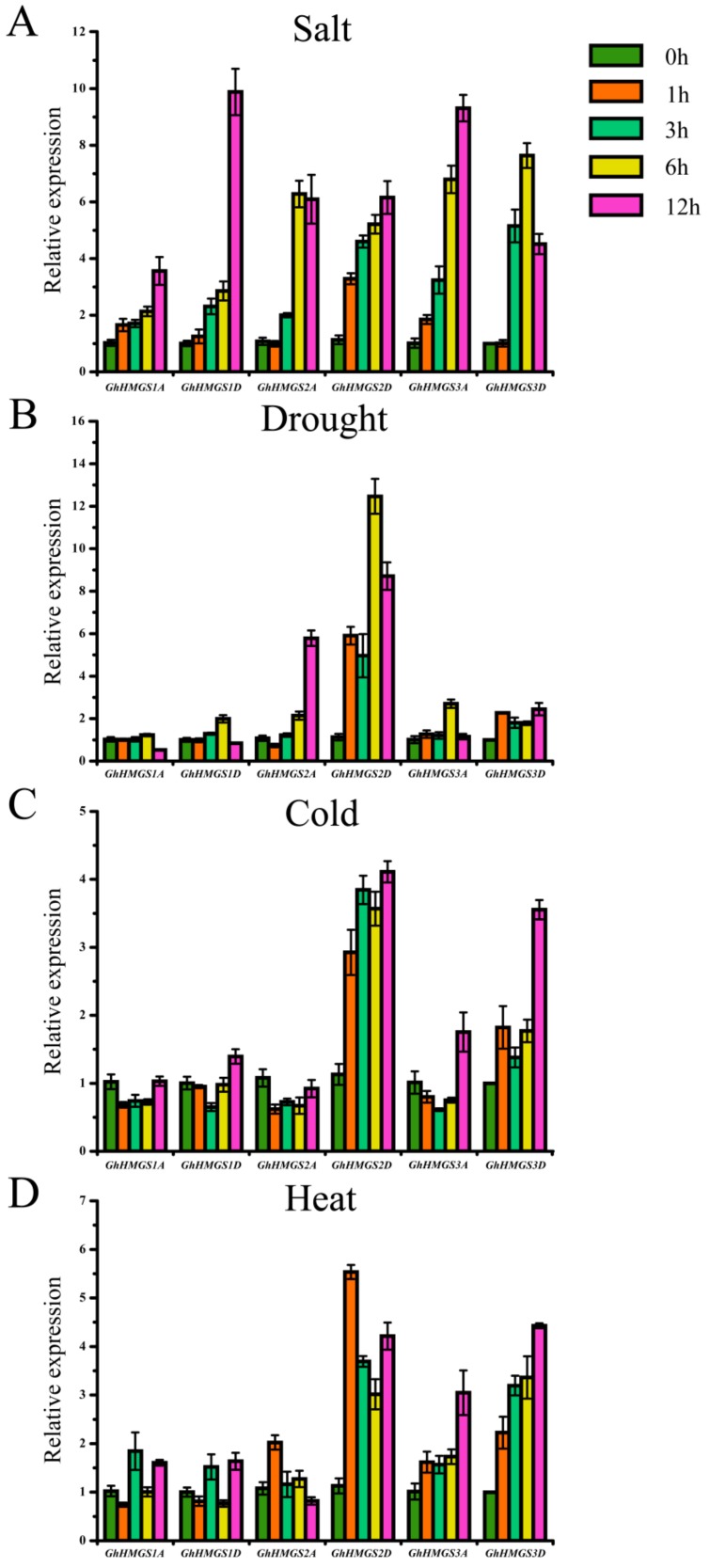
Expression analysis of *G. hirsutum HMGS* genes under abiotic stresses: (**A**) salt; (**B**) drought; (**C**) cold; (**D**) heat. The relative expression levels of *GhHMGS* genes under different abiotic stresses were calculated with the cotton *UBQ7* gene as an internal control. Error bars represent the standard deviations estimated from three independent replicates.

Earlier investigations proved that HMGS is responsive to diverse abiotic stresses, such as exposure to cold, ultraviolet B [35], and drought [34]. These findings imply that HMGS may participate in the resistance of plants to environmental stresses. The expression patterns of *GhHMGS* genes in response to salt, drought, cold, and heat stresses were analyzed (Figure 6). Both *GhHMGS1A* and *GhHMGS1D* were induced by salt stress. Under the drought stress conditions simulated by 20% PEG, *GhHMGS1A* expression was slightly down-regulated at 12 h, but *GhHMGS1D* expression was slightly up-regulated at 6 h before decreasing slightly. When seedlings were exposed to 4 °C for up to 12 h, *GhHMGS1A* expression was down-regulated, while *GhHMGS1D* expression was down-regulated during the first 6 h but was slightly up-regulated at 12 h. *GhHMGS1A* and *GhHMGS1D* exhibited complex expression profiles upon exposure to high temperature (38 °C) conditions. Moreover, *GhHMGS2D* was induced by all four tested abiotic stresses, but *GhHMGS2A* expression was down-regulated under the cold treatment. The expression levels of both *GhHMGS3A* and *GhHMGS3D* were significantly up-regulated under salt stress. Meanwhile, in response to simulated drought stress (20% PEG), *GhHMGS3A* expression was unaffected at the early time points but was up-regulated at 6 h. In contrast, *GhHMGS3D* expression was slightly up-regulated throughout the imposed drought conditions. The cold stress treatment decreased the *GhHMGS3A* expression level during the first 6 h but increased it at 12 h. Meanwhile, *GhHMGS3D* expression was up-regulated and reached a peak at 12 h. In response to heat stress, *GhHMGS3A* and *GhHMGS3D* exhibited similar up-regulated expression profiles, with the highest expression levels at 12 h.

## 4. Discussion

### 4.1. Comparative Genomic Analysis of the HMGS Gene Family in Green Plants

The completion of whole genome sequences of various plant species provides us an opportunity to perform a genome-wide identification and comparative analysis of the *HMGS* genes in green plants. In this study, we first identified 53 *HMGS* genes in 26 different species, including 23 land plant species and three green algae species. The land plant species consisted of 1–5 *HMGS* genes, whereas the examined green algae lacked *HMGS* genes (Figure 1 and Table S4). As the second enzyme in the MVA pathway, HMGS is reportedly a major rate-limiting enzyme during terpene biosynthesis [32]. The MVA pathway is an ancient pathway for the synthesis of terpenes, which are ubiquitous in the three domains of life (bacteria, archaea, and eukaryotes) [51]. The lack of *HMGS* genes in the analyzed green algae implies that the MVA pathway was eliminated in green algae during evolution. Additionally, earlier biochemical experiments also proved that green algae synthesize terpenes only via the MEP pathway [52]. The fact that *HMGS* genes were ubiquitous in the examined land plants suggests that the MVA pathway is still operating in land plants, which is consistent with the previous study [53]. The MVA and MEP pathways were simultaneously retained in land plants, wherein they synthesize specific terpenes in the cytoplasm and plastids, respectively. The retention and compartmentalization of the two pathways may help to balance growth and defense activities to facilitate the survival of plants in dynamic environments [30].

The phylogenetic trees were constructed based on 53 full-length HMGS protein sequences by two different methods (Figure 2 and Figure S1). The phylogenetic analysis indicated that the *HMGS* genes from bryophytes, lycophytes, lycophytes, pteridophytes, gymnosperms, basal angiosperms, monocots, basal eudicots, and core eudicots formed their own evolutionary branches, suggesting that the plant *HMGS* gene may have originated from a common ancestral gene, and the earliest gene family expansion occurred after the divergence of monocots and eudicots. The phylogenetic trees included several species-specific evolutionary branches for *S. lycopersicum*, *P. trichocarpa*, and *G. max*, indicating that a species-specific expansion of *HMGS* gene family took place after the formation of these species.

### 4.2. Evolutionary Conservation of the Cotton HMGS Gene Family

The genome data of the allotetraploid cotton species, *G. hirsutum* [18], and two diploid cotton species, *G. arboreum* [22] and *G. raimondii* [19], are useful for clarifying the evolution of *HMGS* genes during polyploidization events. In this study, we identified three *GaHMGS* genes, three *GrHMGS* genes, and six *GhHMGS* genes. Functional *Gossypium* HMGS proteins contained a conserved motif and active sites similar to other plant HMGS proteins [32]. The phylogenetic analysis revealed one-to-one orthologous relationships between three *GaHMGS* genes and three *GrHMGS* genes. There were twice as many *GhHMGS* genes in the allotetraploid species than in the two diploid species. Moreover, *GrHMGS* and *GaHMGS* genes were distributed on three chromosomes and had one-to-one orthologous relationships with the *HMGS* genes in the *G. hirsutum* D-subgenome and A-subgenome, respectively (Figure 3). The *HMGS* gene family may have expanded in the common progenitor of the two diploid species, after which it was distributed to *G. raimondii* and *G. arboreum* during speciation and then doubled in the allotetraploid species during the process of allopolyploidization. Thus, increases in the number of *HMGS* loci may have been common among cotton genomes.

Gene structure analysis may provide important information relevant to the evolutionary history of gene families [54]. An investigation of the exon–intron structures of *Gossypium HMGS* genes encoding functional proteins revealed that the number and lengths of exons were highly conserved, with the genes generally comprising 12 exons and 11 introns. The homologous genes (*GaHMGS1*, *GhHMGS1A*, *GrHMGS1*, and *GhHMGS1D*; *GaHMGS3*, *GhHMGS3A*, *GrHMGS3*, and *GhHMGS3D*) between diploid and allotetraploid cotton species had almost identical exon–intron structures. The allotetraploid, *G. hirsutum,* likely formed following an interspecific hybridization event between a D-genome species as the pollen parent and an A-genome species as the maternal parent [55]. The similarities in the exon–intron structures and the number of *HMGS* genes in the diploid and allotetraploid species were indicative of a conserved evolution, during which two diploid species hybridized to form an allotetraploid species.

### 4.3. Evolutionary Divergence of the Cotton HMGS Gene Family

In this study, we analyzed and compared the expression profiles of allotetraploid cotton *HMGS* genes in different tissues and in response to various stresses (Figure 4, Figure 5 and Figure 6). Tissue-specific *HMGS* expression patterns were observed, with most genes highly expressed in the roots. The abundance of terpenes such as gossypol and its derivatives in cotton roots is inhibitory toward the growth of pathogenic fungi in the soil [56]. It could be speculated that the high expression level of *HMGS* genes in cotton roots may be related to the considerable demand for the precursors of terpene biosynthesis via the MVA pathway. Plant *HMGS* genes contribute to the response to abiotic stresses and phytohormones [31]. In this study, cotton *HMGS* genes profiles differed under four phytohormone treatments (GA, IAA, SA, and MeJA) and four abiotic stresses (salt, drought, cold, and heat), with most of the gene expression levels being up-regulated. Our findings indicate that *Gossypium HMGS* genes are involved in the signaling pathway regulated by exogenous hormones and the resistance of cotton plants to environmental stresses. 

**Figure 7:**
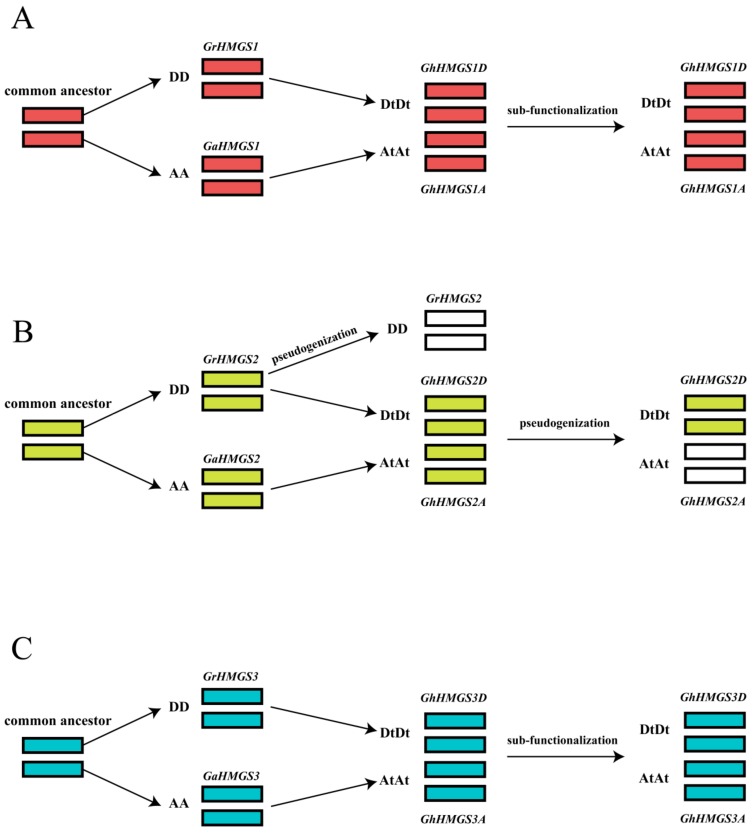
Inferred functional divergence of the duplicated *HMGS* genes in the allotetraploid cotton species, *G. hirsutum*. (**A**) Sub-functionalization of *GhHMGS1A* and *GhHMGS1D*, the red boxes represent genes encoding functional proteins; (**B**) pseudogenization of *GhHMGS2A* and *GhHMGS2D*, the yellow boxes represent genes encoding functional proteins and the white boxes represent the pseudogenes; (**C**) sub-functionalization of *GhHMGS3A* and *GhHMGS3D*, the blue boxes represent genes encoding functional proteins.

Gene duplication plays an extremely important role in the process of biological evolution and is an important source of material for the origin of evolutionary novelties [11]. There are three evolutionary fates of duplicated genes in the genome: (1) neo-functionalization (convert to other functional genes); (2) sub-functionalization (maintain original or similar functions); and (3) pseudogenization (lead to “silencing” of one of the two duplicated copies through mutation) [10]. The diversity in the expression of duplicated genes in various tissues, including similar expression levels in one tissue but different expression levels in another, is suggestive of the sub-functionalization of duplicated genes [57,58]. The alcohol dehydrogenase (*adhA*) genes derived from different parents in cotton (*G. hirsutum*), indicative of sub-functionalization, are reportedly differentially expressed in various organs and under abiotic stress treatments [59]. In the current study, the *GhHMGS1A* and the *GhHMGS1D* expression patterns varied slightly in different tissues. The expression level of *GhHMGS1A* was highest in the roots and stems, whereas *GhHMGS1D* was most highly expressed in the roots and cotyledons. The expression patterns of *GhHMGS3A* and *GhHMGS3D* were basically similar with peak expression levels in the roots and ovules at 40 DPA. In addition, the two copies of *GhHMGS1A/D* and *GhHMGS3A/D* exhibited slightly different expression patterns under phytohormone treatments and abiotic stresses, suggesting the sub-functionalization of the duplicated genes, *GhHMGS1A/D* and *GhHMGS3A/D*, respectively (Figure 7A,C). Sub-functionalization, in which the two copies partition the ancestral function or expression patterns, has a profound impact on the evolution of plants and the formation of new species [60]. The role of sub-functionalization seems to be to preserve duplicated copies by partitioning their expression in response to environmental stress [59]. On the one hand, sub-functionalization protects the redundant cotton *HMGS* genes from being eliminated by natural selection during long-term evolution. On the other hand, the differing expression patterns of homologous *HMGS* genes may enable cotton plants to better cope with various stresses in the natural environments.

Regarding *GhHMGS2A/D*, the insertion of an “A” base at the 800-bp position of the coding region in one homolog, *GhHMGS2A*, resulted in a frameshift mutation as well as the introduction of a premature stop codon (TAA). Therefore, *GhHMGS2A* does not encode a functional protein, namely, pseudogenization (Figure 7B). Duplicated pseudogenes are thought to arise from gene duplications, and one copy of the duplicated genes loses the original protein-coding ability as a result of deleterious mutations. Pseudogenization is considered to be a common evolutionary fate of duplicated genes [61], with studies proving that pseudogenes are widespread in plant genomes. Among the 816 pseudogenes with known origins in rice (*O. sativa* L. ssp. *japonica* cv. Nipponbare), 75% originated from gene duplication events, and 12% are expressed [62]. In our study, although *GhHMGS2A* lacked function at the protein level, it could still transcribe and express, although its expression patterns in the examined tissues differed from those of the homologous gene, *GhHMGS2D*. The expression level of *GhHMGS2A* was also influenced by phytohormone treatments and abiotic stresses. Moreover, multiple sequence alignment revealed an inserted “A” base at the 1085-bp position of the *GrHMGS2* gene in diploid species, as well as an introduced premature stop codon (TGA). These changes may have resulted in a gene that does not encode a functional protein. That is to say, one *HMGS* gene in the diploid and allotetraploid cotton species may have become a pseudogene, although the underlying mechanism may have differed. We previously identified a cotton *HMGR* pseudogene in the A-genome and AD-genome, and we speculated that this pseudogene may have been transferred from wild species to cultivars with the A-genome during domestication and then transferred to the A-subgenome during allopolyploidization [63]. These results combined with those of the current study suggest that in *Gossypium* species, the MVA pathway genes evolved differently.

## 5. Conclusions

Although green algae may lack *HMGS* genes, these genes remain ubiquitous in land plants. Plant *HMGS* genes likely originated from a common ancestral gene, and the earliest gene family expansion event occurred after the divergence of monocots and eudicots. The *HMGS* gene family in two diploid cotton species included three members, and it doubled in size in the allotetraploid cotton during the process of allopolyploidization. The structures of *Gossypium HMGS* genes as well as the encoded proteins were conserved from the diploid to the allotetraploid species. The *HMGS* genes in *G. hirsutum* exhibited tissue-specific expression patterns and were responsive to various phytohormones treatments and abiotic stresses. In addition, the duplicated *HMGS* genes in the allotetraploid cotton species had diverse evolutionary fates, including sub-functionalization and pseudogenization. Overall, these findings elucidated the origin as well as the evolutionary conservation and divergence of the *HMGS* gene family in the allotetraploid cotton species.

## Supplementary Materials

Table S1: Sources of genome data for the non-cotton species analyzed in this study. Table S2: Primers for reverse transcription PCR. Table S3: Primers for quantitative real-time RT-PCR. Table S4: Information regarding the *HMGS* genes in the non-cotton species analyzed in this study. Table S5: The information of *HMGS* genes from *Gossypium*. Figure S1: Phylogenetic tree based on HMGS proteins from *G. raimondii*, *G. arboreum*, and 24 other species. The phylogenetic tree was constructed with the NJ method in the MEGA software with default parameters. The bootstrap analysis was conducted with 1000 replicates. Figure S2: Multiple sequence alignment of the predicted *GrHMGS2*, *GhHMGS2D*, *GaHMGS2*, and *GhHMGS2A* coding sequences. Two red boxes represent the location of the “A” base insertion at the 800-bp and the 1085-bp positions in the coding regions of *GhHMGS2A* and *GrHMGS2*, respectively. Three red asterisks indicate the premature stop codon. Figure S3: Alignment of multiple HMGS amino acid sequences from *G. raimondii*, *G. arboreum*, and *G. hirsutum*. The red box represents the conserved motif, “NxD/NE/VEGI/VDx(2)NACF/YxG”, and the active sites are indicated with a red triangle.
