# Supplementary material for: Evolutionary Conservation and Divergence of Genes Encoding 3-Hydroxy-3-methylglutaryl Coenzyme A Synthase in the Allotetraploid Cotton Species Gossypium hirsutum
Source: Cells. 2019 May 3;8(5):412. doi: 10.3390/cells8050412 (PMC6562921; doi:10.3390/cells8050412)
Supplement: Supplementary file 1 [file cells-08-00412-s001.zip › Table S1.docx]

**Table S1:** Sources of genome data for the non-cotton species analyzed in this study.

| **Species** | **Data sources** |
| --- | --- |
| *Ostreococcus lucimarinus*  *Micromonas pusilla*  *Volvox carteri*  *Physcomitrella patens*  *Selaginella moellendorffii*  *Azolla filiculoides*  *Gnetum montanum*  *Picea abies*  *Amborella trichopoda*  *Brachypodium distachyon*  *Oryza sativa*  *Zea mays*  *Sorghum bicolor*  *Nelumbo nucifera*  *Solanum lycopersicum*  *Vitis vinifera*  *Eucalyptus grandis*  *Medicago truncatula*  *Glycine max*  *Populus trichocarpa*  *Carica papaya*  *Arabidopsis thaliana*  *Theobroma cacao*  **Species** | Ostreococcus lucimarinus version 2.0 (https://phytozome.jgi.doe.gov/pz/portal.html#!info?alias=Org_Olucimarinus)  Micromonas pusilla CCMP1545 version 3.0 (https://phytozome.jgi.doe.gov/pz/portal.html#!info?alias=Org_MpusillaCCMP1545)  Volvox carteri version 2.1 (https://phytozome.jgi.doe.gov/pz/portal.html#!info?alias=Org_Vcarteri)  Physcomitrella patens version 3.3 (https://phytozome.jgi.doe.gov/pz/portal.html#!info?alias=Org_Ppatens)  Selaginella moellendorffii version 1.0 (https://phytozome.jgi.doe.gov/pz/portal.html#!info?alias=Org_Smoellendorffii)  Azolla_asm_v1.1 (ftp://ftp.fernbase.org/Azolla_filiculoides/Azolla_asm_v1.1/)  DRYAD (https://datadryad.org/resource/doi:10.5061/dryad.0vm37.2)  Picea_abies v1.0 (ftp://plantgenie.org/Data/ConGenIE/Picea_abies/)  Amborella trichopoda (assembly AMTR1.0) (https://www.ncbi.nlm.nih.gov/genome/?term=Amborella+trichopoda)  Brachypodium distachyon version 3.1 (https://phytozome.jgi.doe.gov/pz/portal.html#!info?alias=Org_Bdistachyon)  RGAP 7 (http://rice.plantbiology.msu.edu/)  Zea mays version 5b.60 (http://www.maizesequence.org/index.html)  Sorghum bicolor version 3.1.1 (https://phytozome.jgi.doe.gov/pz/portal.html#!info?alias=Org_Sbicolor)  Nelumbo nucifera (assembly Chinese Lotus 1.1) (https://www.ncbi.nlm.nih.gov/genome/?term=Nelumbo+nucifera)  Solanum lycopersicum iTAG2.4 (https://phytozome.jgi.doe.gov/pz/portal.html#!info?alias=Org_Slycopersicum)  Vitis vinifera Genoscope.12X (https://phytozome.jgi.doe.gov/pz/portal.html#!info?alias=Org_Vvinifera)  Eucalyptus grandis version 2.0 (https://phytozome.jgi.doe.gov/pz/portal.html#!info?alias=Org_Egrandis)  Medicago truncatula Mt4.0v1 (https://phytozome.jgi.doe.gov/pz/portal.html#!info?alias=Org_Mtruncatula)  Glycine max version 1.1 (https://phytozome.jgi.doe.gov/pz/portal.html#!bulk?org=Org_Gmax)  Populus trichocarpa version 3.1 (https://phytozome.jgi.doe.gov/pz/portal.html#!info?alias=Org_Ptrichocarpa_er)  Carica papaya ASGPBv0.4 (https://phytozome.jgi.doe.gov/pz/portal.html#!bulk?org=Org_Cpapaya)  TAIR10 (https://www.arabidopsis.org/)  Theobroma cacao version 1.1 (https://phytozome.jgi.doe.gov/pz/portal.html#!info?alias=Org_Tcacao)  **Data sources** |
| *Durio zibethinus* | Durio zibethinus genome (https://www.ncbi.nlm.nih.gov/genome/57226) |
