# Supplementary material for: Evolutionary Conservation and Divergence of Genes Encoding 3-Hydroxy-3-methylglutaryl Coenzyme A Synthase in the Allotetraploid Cotton Species Gossypium hirsutum
Source: Cells. 2019 May 3;8(5):412. doi: 10.3390/cells8050412 (PMC6562921; doi:10.3390/cells8050412)
Supplement: Supplementary file 1 [file cells-08-00412-s001.zip › Table S2.docx]

**Table S2:** Primers for reverse transcription PCR.

| **Gene** | **Forward primer (5'-3')** | **Reverse primer (5'-3')** |
| --- | --- | --- |
| *GrHMGS1*  *GrHMGS2*  *GrHMGS3*  *GaHMGS1*  *GaHMGS2*  *GaHMGS3*  *GhHMGS1A*  *GhHMGS1D*  *GhHMGS2A*  *GhHMGS2D*  *GhHMGS3A*  *GhHMGS3D* | ATGGCTAAGAATGTGGGAATTCTTG  ATGGCAAAGAATGTGGGAGTTC  ATGGCTAAGAATGTAGGAATTCTTG  ATGGCTAAGAATGTGGGAATTCTTG  ATGGCAAAGAATGTGGGAGTTCTAG  ATGGCTAAGAATGTGGGAATTATTG  ATGGCTAAGAATGTGGGAATTCTTG  ATGGCTAAGAATGTGGGAATTCTTG  TTTTGTATTGTTGACATAGCGGTTC  ATGGCAAAGAATGTGGGAGTTCTAG  ATGGCTAAGAATGTGGGAATTATTG  ATGGCTAAGAATGTAGGAATTCTTG | CTAATGACCGTTGGCAACGGAAC  TCATGGAGTCGCAATGAAAACA  TCAGTGACCATTGACAACAGAAC  CTAATGACCGTTGGCAATGGAAC  TCAATGTCCGTTAGCAAGGGATC  TCAGTGACCATTGACAACAGAAC  CTAATGACCGTTGGCAATGGAAC  CTAATGACCGTTGGCAACGGAAC  ACATGGAGATAACGCATCTTAGCAC  TCAATGTCCGTTAGCAAGAGATC  TCAGTGACCATTGACAACAGAAC  TCAGTGACCATTGACAACAGAAC |
