# Supplementary material for: Evolutionary Conservation and Divergence of Genes Encoding 3-Hydroxy-3-methylglutaryl Coenzyme A Synthase in the Allotetraploid Cotton Species Gossypium hirsutum
Source: Cells. 2019 May 3;8(5):412. doi: 10.3390/cells8050412 (PMC6562921; doi:10.3390/cells8050412)
Supplement: Supplementary file 1 [file cells-08-00412-s001.zip › Table S3.docx]

**Table S3:** Primers for quantitative real-time RT-PCR.

| **Gene** | **Forward primer (5'-3')** | **Reverse primer (5'-3')** |
| --- | --- | --- |
| *GhHMGS1A*  *GhHMGS1D*  *GhHMGS2A*  *GhHMGS2D*  *GhHMGS3A*  *GhHMGS3D*  *UBQ7* | ATCCCTTATTCACATCAAACAC  ATCCCTTATTCACAACAAAAAC  ACAGGAAGCATTGGAGACTCGT  ACAGGAAGCATTGGAGGCTCAT  ATGTTGCTGGAAAATTGAAGGC  ATTGCTGGAAAATTGAAGTCTA  GAAGGCATTCCACCTGACCAAC | TGCCTTGACTTCAATTTCCCAC  TGTTCGATAGGCTAAACGGGTG  TCAAGGAGTGAAGTAACAGCAG  TTCAAGGAGTGAAGTGACAGCT  TCCGGGAGACAACAGGCTAGAG  CCGGGAGACAAAAGGCTACAAT  CTTGACCTTCTTCTTCTTGTGCTTG |
