# Supplementary material for: Evolutionary Conservation and Divergence of Genes Encoding 3-Hydroxy-3-methylglutaryl Coenzyme A Synthase in the Allotetraploid Cotton Species Gossypium hirsutum
Source: Cells. 2019 May 3;8(5):412. doi: 10.3390/cells8050412 (PMC6562921; doi:10.3390/cells8050412)
Supplement: Supplementary file 1 [file cells-08-00412-s001.zip › Table S4.docx]

**Table S4:** Information regarding the *HMGS* genes in the non-cotton species analyzed in this study.

| **Lineage** | **Species** | **Gene name** | **Gene locus** | **Chromosome** | | **Start** | **End** |
| --- | --- | --- | --- | --- | --- | --- | --- |
| Green algae  Bryophytes  Lycophytes  Pteridophytes  Gymnosperms  Basal angiosperms  Monocots | *Chlorella variabilis*  *Physcomitrella patens*  *Selaginella moellendorffii*  *Azolla filiculoides*  *Gnetum montanum*  *Picea abies*  *Amborella trichopoda*  *Brachypodium distachyon*  *Oryza sativa*  *Zea mays*  *Sorghum bicolor* | *CvHMGS*  *PpHMGS1*  *PpHMGS2*  *SmHMGS1*  *SmHMGS2*  *AfHMGS1*  *AfHMGS2*  *GmoHMGS*  *PbHMGS1*  *PbHMGS2*  *AtrHMGS*  *BdHMGS1*  *BdHMGS2*  *OsHMGS1*  *OsHMGS2*  *OsHMGS3*  *ZmHMGS1*  *ZmHMGS2*  *ZmHMGS3*  *SbHMGS1*  *SbHMGS2*  *SbHMGS3* | IGS.gm_2_00625  Pp3c5_26660V3.1  Pp3c6_5280V3.1  179152  121372  Azfi_s0163.g054193  Azfi_s0232.g059323  TnS000989535t03  MA_101113g0020  MA_261222g0010  XP_011628383.1  Bradi1g77290  Bradi3g42010  LOC_Os03g02710  LOC_Os08g43170  LOC_Os09g34960  GRMZM2G087207  AC199782.5_FG002  GRMZM2G106263  Sobic.001G530100  Sobic.002G268700  Sobic.007G182000 | scaffold_2  Chr05  Chr06  scaffold_51  scaffold_65  Azfi_s0163  Azfi_s0232  scaffold989535  MA_101113  MA_261222  NW_006500346.1  Bd1  Bd3  Chr3  Chr8  Chr9  1  2  9  Chr01  Chr02  Chr07 | | 2260770  18827205  3062792  244914  239125  89941  348796  247609  12449  1512  2691523  73898529  43546666  1015290  27302233  20367318  4011889  195008292  154290417  79386771  65264071  61503627 | 2264230  18831928  3067646  247246  241071  96070  353270  254719  22410  2111  2700952  73904100  43551395  1020208  27307731  20371359  4016576  195011506  154296012  79391901  65267941  61507409 |
| **Lineage** | **Species** | **Gene name** | **Gene locus** | | **Chromosome** | **Start** | **End** |
| Basal Eudicots  Core Eudicots | *Nelumbo nucifera*  *Arabidopsis thaliana*  *Carica papaya*  *Glycine max*  *Eucalyptus grandis*  *Populus trichocarpa*  *Vitis vinifera*  *Durio zibethinus*  *Theobroma cacao*  *Medicago truncatula*  *Solanum lycopersicum* | *NnHMGS1*  *NnHMGS2*  *AthHMGS*  *CpHMGS*  *GmaHMGS1*  *GmaHMGS2*  *GmaHMGS3*  *GmaHMGS4*  *GmaHMGS5*  *EgHMGS*  *PtHMGS1*  *PtHMGS2*  *PtHMGS3*  *VvHMGS1*  *VvHMGS2*  *DzHMGS1*  *DzHMGS2*  *DzHMGS3*  *TcHMGS1*  *TcHMGS2*  *MtHMGS1*  *MtHMGS2*  *SlHMGS1*  *SlHMGS2*  *SlHMGS3* | XP_010255571.1  XP_010272584.1  AT4G11820  evm.model.supercontig_29.22  Glyma01g42450  Glyma09g27380  Glyma11g02950  Glyma16g32480  Glyma17g14150  Eucgr.D01931  Potri.001G111700  Potri.003G120300  Potri.003G120400  GSVIVT01019875001  GSVIVT01010658001  XM_022903921  XM_022864049  XM_022880424  Tc03v2_t013870  Tc03v2_t022490  Medtr5g011040  Medtr6g089570  Solyc08g007790  Solyc08g080170  Solyc12g056450 | | NW_010729088.1  NW_010729149.1  Chr4  supercontig_29  Gm01  Gm09  Gm11  Gm16  Gm17  Chr04  Chr01  Chr03  Chr03  chr2  chr16  NW_019167827.1  NW_019167838.1  NW_019168381.1  chr3  chr3  chr5  chr6  SL2.50ch08  SL2.50ch08  SL2.50ch12 | 6620004  121659  7108915  215266  53696165  34191078  1927111  35624252  10913002  32817238  8907838  14265134  14282609  4131461  15476652  2094935  3427900  3382548  28181808  33628762  3104283  22740317  2294082  63490531  62391253 | 6628378  130832  7112480  221239  53701779  34197302  1933660  35630761  10917928  32822915  8912053  14269761  14285782  4138394  15482836  2100224  3432664  3388405  28186623  33633443  3109011  22746750  2299193  63495072  62395143 |
