# Supplementary material for: Evolutionary Conservation and Divergence of Genes Encoding 3-Hydroxy-3-methylglutaryl Coenzyme A Synthase in the Allotetraploid Cotton Species Gossypium hirsutum
Source: Cells. 2019 May 3;8(5):412. doi: 10.3390/cells8050412 (PMC6562921; doi:10.3390/cells8050412)
Supplement: Supplementary file 1 [file cells-08-00412-s001.zip › Table S5.docx]

**Table S5:** The information of *HMGS* genes from *Gossypium*.

| **Species** | **Gene name** | **Gene locus** | **Chromosome** | **Location** | **Strand** | | **Protein length** | | **Mw (kDa)^a^** | **pI^a^** |
| --- | --- | --- | --- | --- | --- | --- | --- | --- | --- | --- |
| *G. raimondii*  *G. arboreum*  *G. hirsutum* | *GrHMGS1^b^*  *GrHMGS2^c^*  *GrHMGS3*  *GaHMGS1*  *GaHMGS2*  *GaHMGS3*  *GhHMGS1A^b^*  *GhHMGS1D*  *GhHMGS2A^bc^*  *GhHMGS2D*  *GhHMGS3A*  *GhHMGS3D* | Gorai.003G163200  Gorai.004G207000  Gorai.008G243100  evm.model.Ga01G2629 evm.model.Ga08G2094  evm.model.Ga12G0545 Ghir_A03G002030  Ghir_D03G016950  Ghir_A08G018570  Ghir_D08G019470  Ghir_A12G023770  Ghir_D12G023770 | Gr_Chr3  Gr_Chr4  Gr_Chr8  Ga_Chr1  Ga_Chr8  Ga_Chr12  Gh_At_Chr3  Gh_Dt_Chr3  Gh_At_Chr8  Gh_Dt_Chr8  Gh_At_Chr12  Gh_Dt_Chr12 | 43251375-43255184  53750818-53754952  52847645-52851867  110496438-110499725  118882851-118886489  4709389-4713086  3081088-3084380  49811535-49815847  112536471-112540700  59658654-59663182  102705221-102709478  57871193-57875545 | +  −  −  +  −  +  +  +  −  −  −  − | 465  _  465  465  465  465  465  465  _  465  465  465 | | 51.32  _  51.48  51.34  51.65  51.45  51.32  51.28  _  51.68  51.42  51.52 | | 6.43  _  6.33  6.47  6.28  6.33  6.47  6.43  _  6.15  6.20  6.20 |

^a^ The theoretical Mw (molecular weight) and pI (isoelectric point) of the full-length protein are predicted by ProtParam tool (http://web.expasy.org/protparam/). ^b^ The coding sequences of genes are re-annotated. ^c^ The gene is identified as a pseudogene.
