# Supplementary figures and images for: Evolutionary Conservation and Divergence of Genes Encoding 3-Hydroxy-3-methylglutaryl Coenzyme A Synthase in the Allotetraploid Cotton Species Gossypium hirsutum
Source: Cells. 2019 May 3;8(5):412. doi: 10.3390/cells8050412 (PMC6562921; doi:10.3390/cells8050412)

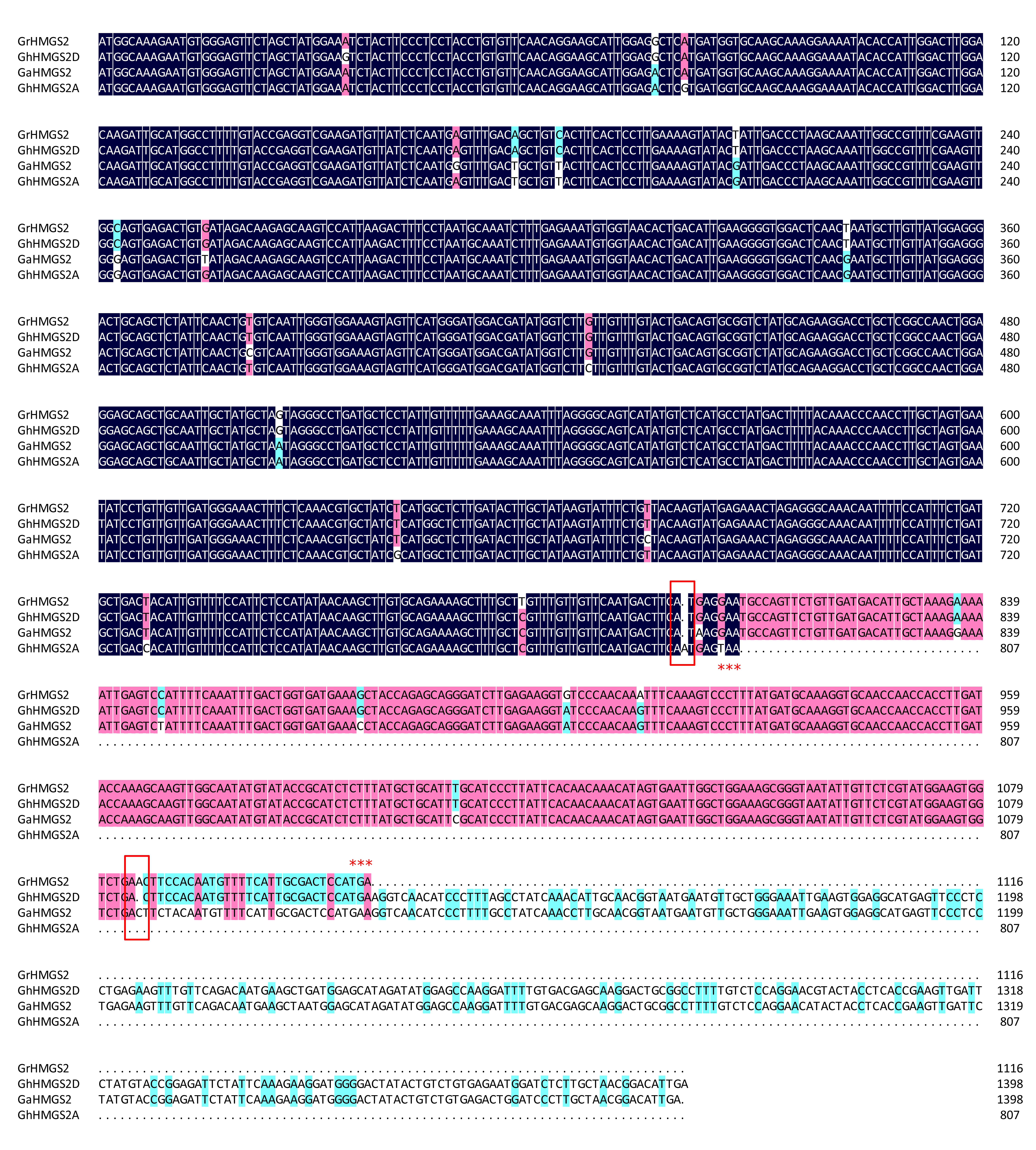

Supplement: Supplementary file 1 [file cells-08-00412-s001.zip › Figure S2.tif]
